# Testing and evaluation of lower limb prosthesis prototypes in people with a transfemoral amputation: a scoping review on research protocols

**DOI:** 10.1186/s12984-023-01125-8

**Published:** 2023-01-12

**Authors:** Vera G. M. Kooiman, Eline S. van Staveren, Ruud A. Leijendekkers, Jaap H. Buurke, Nico Verdonschot, Erik C. Prinsen, Vivian Weerdesteyn

**Affiliations:** 1 Orthopedic Research Laboratory, Radboud University Medical Center, P.O. Box 9101, 6500 HB Nijmegen, The Netherlands; 2 Department of Rehabilitation, Radboud University Medical Center, Donders Institute for Brain, Cognition and Behaviour, P.O. Box 9101, 6500 HB Nijmegen, The Netherlands; 3 Roessingh Research and Development, PO Box 310, 7500 AH Enschede, The Netherlands; 4 Radboud Institute for Health Sciences, IQ Healthcare, Radboud University Medical Center, P.O. Box 9101, 6500 HB Nijmegen, The Netherlands; 5 Roessingh Center for Rehabilitation, Postbus 310, 7500 AH Enschede, The Netherlands; 6 Department of Biomechanical Engineering, Faculty of Engineering Technology, University of Twente, P.O. Box 217, 7500 AE Enschede, The Netherlands; 7 Sint Maartenskliniek, Research & Rehabilitation, P.O. Box 9011, 6500 GM Nijmegen, The Netherlands

**Keywords:** Prosthetics, Development, Prototype, Testing, Protocol, Transfemoral, Evaluation, Design

## Abstract

**Background:**

When developing new lower limb prostheses, prototypes are tested to obtain insights into the performance. However, large variations between research protocols may complicate establishing the potential added value of newly developed prototypes over other prostheses.

**Objective:**

This review aims at identifying participant characteristics, research protocols, reference values, aims, and corresponding outcome measures used during prosthesis prototype testing on people with a transfemoral amputation.

**Methods:**

A systematic search was done on PubMed and Scopus from 2000 to December 2020. Articles were included if testing was done on adults with transfemoral or knee disarticulation amputation; testing involved walking with a non-commercially available prototype leg prosthesis consisting of at least a knee component; and included evaluations of the participants’ functioning with the prosthesis prototype.

**Results:**

From the initial search of 2027 articles, 48 articles were included in this review. 20 studies were single-subject studies and 4 studies included a cohort of 10 or more persons with a transfemoral amputation. Only 5 articles reported all the pre-defined participant characteristics that were deemed relevant. The familiarization time with the prosthesis prototype prior to testing ranged from 5 to 10 min to 3 months; in 25% of the articles did not mention the extent of the familiarization period. Mobility was most often mentioned as the development or testing aim. A total of 270 outcome measures were identified, kinetic/kinematic gait parameters were most often reported. The majority of outcome measures corresponded to the mobility aim. For 48% of the stated development aims and 4% of the testing aims, no corresponding outcome measure could be assigned. Results indicated large inconsistencies in research protocols and outcome measures used to validate pre-determined aims.

**Conclusions:**

The large variation in prosthesis prototype testing and reporting calls for the development of a core set of reported participant characteristics, testing protocols, and specific and well-founded outcome measures, tailored to the various aims and development phases. The use of such a core set can give greater insights into progress of developments and determine which developments have additional benefits over the state-of-the-art. This review may contribute as initial input towards the development of such a core set.

**Supplementary Information:**

The online version contains supplementary material available at 10.1186/s12984-023-01125-8.

## Background

People using a lower limb prosthesis face additional challenges during daily life in comparison to able-bodied persons. Performing gait-related task requires more metabolic energy and cognitive effort, and their gait is less stable [1–7]. Gait-related tasks are specifically demanding for people with a transfemoral amputation, due to the lack of an active knee joint. This requires people with a transfemoral amputation to enhance the control and power of other body parts during walking in comparison to people with a transtibial amputation [2, 8–10]. To assist persons with a transfemoral amputation in overcoming these difficulties, significant progress has been made in the development of lower limb prostheses.

As with all new developments, it is essential to test the product on potential benefits before determining whether to pursue making it commercially available. Prototype testing provides insights into the performance of the product and its potential added value for the patient over other state-of-the-art prosthesis. However, presently, there are no standardized methods on how to evaluate the performance and functionality of a pre-commercial prosthesis prototype or prosthetic parts during walking. Consequently, researchers need to draft their own testing protocols to evaluate the aims set for the development of different types of prosthesis prototype. This could in turn lead to large variations between the testing protocols of the different prototypes. Large variations between testing protocols restricts comparing testing results, which complicates interpreting the potential added value of the developed prototype over other prostheses (commercially as well as non-commercially available ones). The large variations in testing protocol and outcome measures has been demonstrated in efficacy testing of lower limb prostheses [11]. However currently, an overview is lacking of the study population and the testing protocols used to validate specific development or testing aims, the respective outcome measures of interest, and the values to be used as a reference for evaluating prosthesis prototypes.

Therefore, this review aimed to provide a comprehensive overview of participant characteristics reported, research protocols, reference values, and outcome measures that are used during prosthesis prototype testing on people with a transfemoral amputation. In addition, it will be evaluated whether the reported outcome measures corresponded to specific development or testing aims. The information provided by this review can be a starting point for future harmonization of prosthesis testing and development of standardized testing methods, which allows for better comparison of research outcomes across studies and supports in quantifying the progress in lower limb prosthesis development.

## Method

### Search strategy

An electronic search was done via PubMed and Scopus from January 2000 to December 2020. Articles published before 2000 were excluded from the search to avoid inclusion of obsolete measurement technologies used during the evaluations or obsolete prosthesis prototypes being evaluated. The search strategy was based on the following three terms:

[1] (gait [tiab] OR walk* [tiab])

[2] (transfemoral [tiab] OR above-knee[tiab])

[3] (prosthes* [tiab] OR “artificial limb” [tiab] OR "Artificial Limbs"[Mesh] OR amput* [tiab] OR "Amputation"[Mesh])

As different terminology is used in scientific literature regarding the testing of non-commercial prototypes, the search strategy was not further specified in this aspect. As this is a scoping review the research protocol was not registered in a database of prospectively registered reviews.

### Selection criteria

The first screening was done based on title and abstract of each individual study by two reviewers independently. Studies were included in the full text review when the following criteria were met:

1. Testing was done on a non-commercially available prototype leg prosthesis (at time of testing) consisting of at least a knee component.
2. Testing was focused on adults with a transfemoral amputation or knee disarticulation.
3. Testing was done at least during level walking (overground or on a treadmill).
4. Testing results had to include evaluations of functioning of the participant when walking with the prosthesis prototype.

If the full-text version of the article was available, an extended abstract of the same research was considered a duplicate. If not, extended abstracts were included. Articles written in other languages than English were excluded. Full-text articles were assessed by two reviewers independently using the same criteria.

### Data extraction

Data extracted from the included studies included: participant characteristics, prosthesis (prototype) specifications, research protocol, reference value, outcome measures, and aims as defined by the article. Participant characteristics included: number of participants, sex, age, amputation level, reason of amputation, level of activity, and current prosthesis. For both the participants current prosthesis and the prosthesis prototype it was noted whether the knee and ankle component were passive (P), micro-processor controlled (MPC), or powered (PW). For the research protocol, the familiarization method with the prototype prosthesis, the tasks executed during testing, and the chosen walking speed were extracted, as well as the outcome measures and the reference value used to evaluate the prosthesis prototype. In addition, the aims set for both the development and testing of the prosthesis prototype with potential end-users were registered and categorized. Definitions of the aim categories are displayed in Table 1. The aims were categorized into the following categories: Subjective evaluation; Mobility; Energy consumption of participant; Energy handling of prosthesis prototype; Prosthesis control; Effect of powered prosthesis; and Prototype characteristics. Operationalization of the aim categorization can be found in Additional file 1: Table S1.

**Table 1:** Definition of the aim categories

Aim category	Definition
Subjective evaluation	Subjective aims regarding participant’s or physiotherapist’s perspective after user-trials, like an interview
Mobility	Aims regarding mobility, for example walking ability or gait assessment
Energy consumption of participant	Aims regarding metabolic cost and energy consumption of participants when walking with the prosthesis prototype
Energy handling of prosthesis prototype	Aims regarding energy handling of the prosthesis prototype, for example battery life, battery recharge during gait, or energy efficiency
Prosthesis control	Aims regarding the control algorithm of the prosthesis prototype, for example providing swing or stance phase control, control simplicity, intent recognition, algorithm accuracy, or delays
Effect of powered prosthesis	Aims including the delivery and output of the required power of the prosthesis, for example power generation similar to an anatomical joint, providing power during climbing the stairs, or inject small amounts of power during the swing phase
Prototype characteristics	Aims on the characteristics and properties of the prosthesis prototype, for example device weight, size, cultural, and aesthetic aspects, or production cost

Likewise, outcome measures were assigned to 1 of 9 separate self-defined categories: Clinical & qualitative measures; Spatiotemporal; Kinetic/kinematic; Metabolic energy; Muscle activity; Prosthesis control; Electrical (prosthesis) energy; Prototype characteristics; and Other.

A graphical overview was created showing development and testing aims, and their corresponding outcome measures. For this purpose, the outcome measures of each article were linked to their respective aims. Remaining outcome measures, which could not be linked to a development or testing aim, were added to a ‘miscellaneous’ category.

Article selection, data extraction and categorization were executed independently and in duplicate by two reviewers. Discrepancies were discussed until an agreement was reached. Data were organized in tables according to Preferred Reporting Items for Systematic Reviews and Meta-Analyses (PRISMA) extension for scoping reviews [12].

## Results

### Data selection

**Fig. 1:**
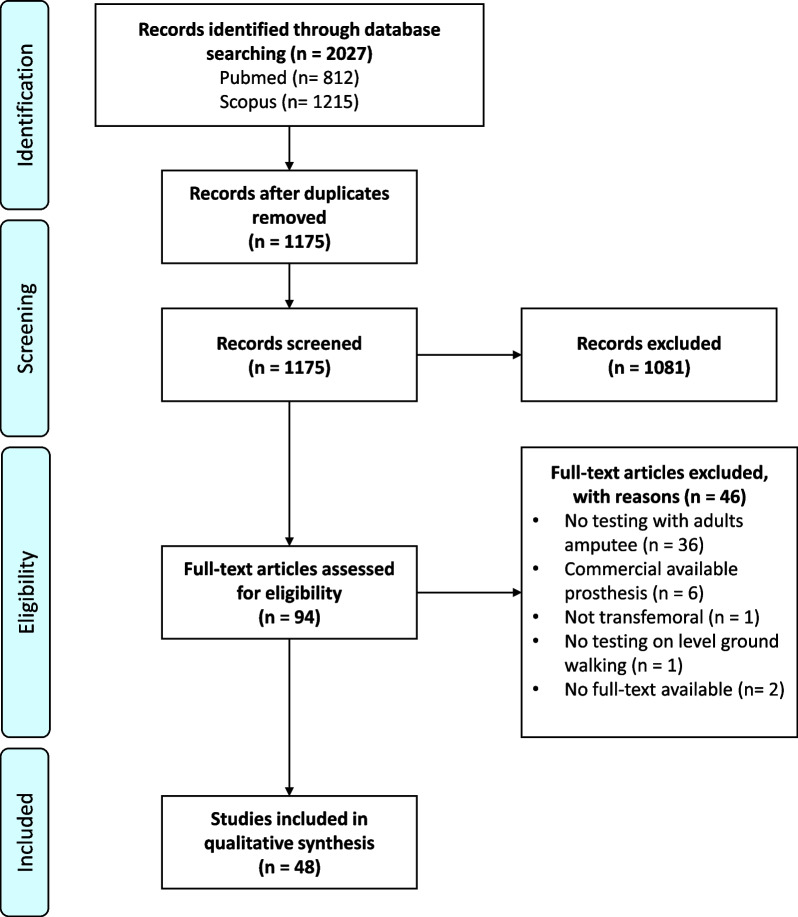
PRISMA extension for scoping reviews flow diagram of the article selection process

The initial search resulted in 2027 titles, of which 94 studies were selected for full-text screening (Fig. 1). Of these full-text articles, 36 articles were excluded because these did not concern adults with an amputation (e.g., used benchmark or able-bodied adaptor testing), six articles studied commercially-available prostheses, one article did not involve participants with a transfemoral amputation, one article did not test on level walking, and two articles were not available in full text. The remaining 48 articles were included in this review [13–60].

### Participant characteristics and research protocol

Data extracted from the articles on participant characteristics and the research protocol are summarized in Additional file 2: Table S2. Of the 48 selected articles, 20 studies were single-subject studies and 4 studies included a cohort of 10 or more persons with a transfemoral amputation. Additional measurements on able-bodied persons as reference values were conducted in 5 studies (n = 16), of which one study actually tested the prototype using an able-bodied adapter (n = 1). Of all participants (n = 152), 59% were identified as male (n = 89), 7% as female (n = 11), and sex was unspecified in 35% (n = 52). The age of the participants ranged from 15 to 75 years. Trauma was the most frequently reported reason for amputation (50 of 136 people with an amputation), while 32 articles did not report the reason for amputation (n = 65). Twelve of the 18 articles that reported the level of activity defined their participants as highly active (i.a. K3 or higher). The remaining six articles reported a lower level of activity (i.a., K1 or K2). Of the 48 included articles, 28 specified the current knee prosthetic part used by the participants, and 9 specified the current ankle prosthetic part.

In the research protocols, familiarization time was specified in 32 articles, whereas 4 articles did not report a specific duration, and 12 articles did not report whether participants were given any familiarization time. The familiarization time reported in the articles ranged from 5 to 10 min to 3 months. A familiarization time of less than one day was reported in 19 articles, with the majority (16 articles) reporting a familiarization time of less than one hour. For evaluating the prosthesis prototype, 26 studies used walking overground as the experimental task, whereas treadmill walking was used in 24 studies; 2 studies used both. Thirty studies used a self-selected comfortable walking speed, whereas multiple speed levels were used in 16 studies. For comparing the outcomes of interest, 18 articles used reference values derived from recordings performed with the amputee’s current prosthesis, 11 articles used reference values from literature, and 8 articles used the intact limb of the amputee as a reference. Further comparisons were described between walking speeds (n = 5), between settings (n = 4), with measurements from able-bodied persons (n = 3), with desired trajectories (n = 4), and with sensor data (n = 1). Five articles reported results without any comparison of their outcome measures.

### Article aims

From all articles, 145 development and testing aims were identified and categorized according to the definition in Table 1 (see Additional file 1). In the majority of the articles (80%), more than one development or testing aim was identified. The development and testing aim of improving mobility was most often mentioned in the articles. This was the case in 63% and 60% of the 48 articles for the development and testing aims, respectively. Other frequently mentioned aims included the effect of powered prosthesis aim (35%) and prototype characteristics aim (33%), both only mentioned as development aim. Consecutively, the prosthesis control, energy consumption regarding participant, and energy handling of prosthesis prototypes aims were mentioned in 15–27% and 6–15% of the articles as the development and testing aims, respectively. Aims focusing on subjective evaluation (4% development aim, 6% testing aim) were minimally represented. Of all articles, 8% and 27% did not define any specific development or testing aim, respectively.

### Outcome measures

Of all outcome measures (n = 270), kinetic/kinematic gait parameters were most often reported (50%), followed by the spatiotemporal gait parameters (11%). The most frequently reported outcome measure overall was the knee angle (degrees) displayed over the gait cycle (13%), mainly corresponding to the mobility aim.

**Fig. 2:**
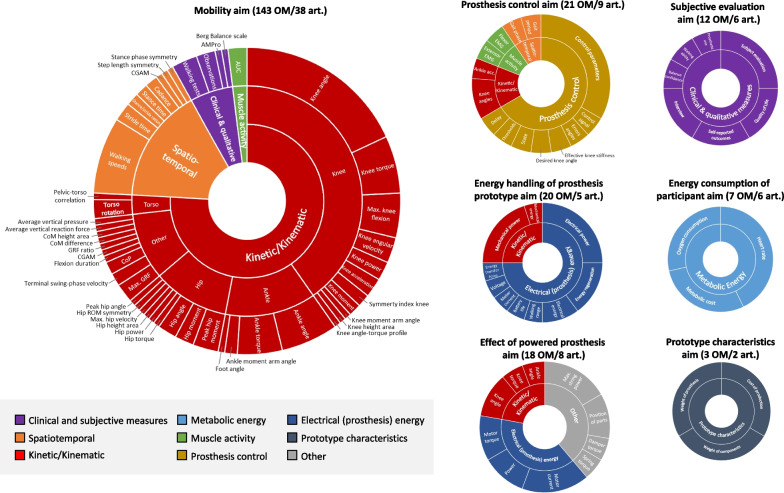
Visual overview of the aims with their corresponding outcome measures and outcome measures categories. OM., amount of outcome measures in the aim; art., amount of articles with an outcome measure that corresponded to the aim. acc., acceleration; AUC, area under the curve; CGAM, combined gait asymmetry metric; CoM, centre of mass; CoP, centre of pressure; EMG, electromyography; GRF, ground reaction force; ROM, range of motion

The overview of the combined development and testing aims with their corresponding outcome measures can be found in Fig. 2. Excluded from these figures were the aims where no corresponding outcome measure could be assigned. This was the case in 48 of the 145 aims stated (48% of the development aims, 4% of the testing aims). In addition, 45 (17%) of the 270 outcome measures reported could not be assigned to a corresponding aim and were added to the miscellaneous category.

The majority of the studies focused on the mobility aim with more than half of all identified outcome measures corresponding to this aim (51%); 83% of these outcome measures involved kinetic and kinematic parameters. For the prosthesis control aim, the most commonly reported outcome measures were the control parameters (33%); for the energy handling of prosthesis prototype aim this was the electrical power (25%); for the effect of powered prosthesis aim this were the maximal string power and motor current (both 17%); for the subjective evaluation aim this was the subject evaluation (25%); and for the energy consumption of the participant aim this was the heart rate (43%). For the prototype characteristics aim, all outcome measures were mentioned equally often (33%). The outcome measures in the miscellaneous category (i.e. where no corresponding aim was defined in the article) included prosthesis control (26%), kinetic/kinematic (22%), electrical (prosthesis) energy (17%), other (15%), clinical & qualitative measures (7%), spatiotemporal (7%), and metabolic energy (7%) outcomes. The reference list in Additional file 3 (Table S3) shows which articles contain the corresponding outcome measures for each of the aim categories.

## Discussion

This review aimed to provide an overview of studies that reported on pre-commercial transfemoral prosthesis prototype evaluations during walking. Given the continuous development of new prosthesis prototypes and the desire to systematically assess their functional benefits, this review summarized the participant characteristics reported as well as the research protocols, reference values, and outcome measures used. In addition, results were presented in a graphical overview of the aims with their corresponding outcome measures.

Among the included articles, the participants’ characteristics were reported to a highly varying extent. Notably, a mere 5 articles reported all our pre-defined participant characteristics; the characteristic that was least frequently reported was the specification of the participants’ prosthetic foot. The reported characteristics indicated that a majority of participants were active males with a traumatic amputation, which is not representative of the population of persons with an amputation at large [61, 62]. The age of the participants varied greatly, ranging from 15 to 75 years. Giving a thorough description of the study population is important for making inferences on the external validity of the study findings, i.e. to help judge whether the observed performance of a prosthesis prototype can be generalized to a larger population of persons using a lower-limb prosthesis. The performance of a prosthesis prototype may be different for e.g., active persons with a traumatic amputation or less active persons with a vascular amputation. The use of a minimal reporting set of participant characteristics should therefore be agreed upon, which can be expanded depending on participant characteristic that are specifically related to development or testing aims.

In general, the research protocol was quite elaborately described in the included articles, although in 25% of the articles there was no information given about the familiarization period to the prosthesis prototype. In addition, in the studies that specified the familiarization period, there was a large variety in the reported duration of the familiarization period, which ranged from 5 to 10 min to 3 months. This large variation may (at least partly) be related to the development stage of the prototype (technology readiness level, TRL) in which testing took place. In the current review, all stages of pre-commercial prosthesis prototype testing were included. In early stages of development, a prosthesis prototype commonly lacks a CE (Conformité Européenne) or FDA (Food and Drug Administration) certification. The lack of such certification prevents its unsupervised use at home, which restricts extensive familiarization to fully adapt to the prosthesis prototype. Yet, when testing is done in the early TRL stages of a new prosthesis prototype, a limited familiarization period may suffice for evaluating the development aims, whereas it is conceivable that in later TRL stages, more extensive familiarization periods are required to allow for sound conclusions on the participants’ walking performance with the prosthesis prototype in daily life. Furthermore, the targeted study population may also influence the amount of adaptation time needed [63–65]. Familiarization time may therefore be adjusted to the study population, aims set for the testing as well as the TRL stage of the prosthesis prototype. However, it should be noted that limited literature is available on motor learning and adaptation when receiving a different prosthesis. Additional research is needed to adequately determine the required familiarization time to fully adjust to walking with a new prosthesis or prosthetic part, for instance taking into account age, level of activity and cause of amputation.

The reference values used for comparing the outcome measures varied across the included articles, which may also be related to the different TRL stages of the prosthesis prototype tested. The included articles most commonly used the amputee’s current prosthesis and literature values as a reference. During the initial design and development process of a prosthesis prototype, reference values as reported in the literature are typically used in defining the design. Therefore, using literature values for comparison of the testing results may suffice for validating the design in the early TRL stages. Yet, once the stage is reached that testing aims involve performance in daily life, it is important to study the potential benefits of the prosthesis prototype as compared to the participant’s own state-of-the-art prosthesis. Accordingly, the reference values, as well as outcome measures used may be adapted to the testing aims set by the researchers, with an increasing need towards assessing performance in daily life when the prototype is in a higher TRL stage.

Regarding the development and testing aims of the included articles, the mobility aim was most frequently mentioned. It was associated with the majority of the reported outcome measures, which might be inherent to the inclusion criteria of human-in-the-loop evaluations during gait. In the majority of the included articles, the reported outcome measures could be linked to the stated testing aims, whereas a large part of the development aims did not have associated outcome measures. From the descriptions in the included articles, it appears that the design and development of a new prosthesis prototype often include multiple aims, while the reported testing results are often focused on one aim at a time [16–19, 21, 22, 25, 26, 33, 37, 39, 40, 42–44, 46, 47, 57]. Nonetheless, other aims stated for the development of the prosthesis prototype may be tested and reported in follow-up articles, like in the case of CYBERLEG [13, 24–26, 66]. However, it should also be noted that a testing aim was not explicitly formulated in 27% of the articles, while a development aim remained unspecified in 8% of the articles. Formulating an explicit testing aim facilitates comparing the testing results to other research with similar testing aims.

Overall, the presented overview of the literature on transfemoral prosthesis prototype testing shows that there is large heterogeneity in research protocols and outcome measures used to evaluate the stated aims. No consistency could be found in the outcome measure(s) for evaluating certain aims. Especially for the mobility aim, a substantial number of different outcome measures were used. In addition, a justification of the selected outcome measures was often not given in the articles, which complicates putting the results into perspective in comparison to those reported for other prosthesis prototypes or commercially-available prostheses. A justification of the selected outcome measures may help determine the added value of the innovative designs of the prosthesis prototypes over the state-of-the-art. Therefore, it is recommended that all articles regarding prosthesis prototype testing should clearly state the development aim of the prototype, the testing aim of the article, and which outcome measures were selected to evaluate defined testing aim.

The field of transfemoral prostheses development and research is rapidly advancing, and this article presents the first comprehensive review summarizing the literature on prosthesis prototype testing that was published in the past two decades. As there was no established framework for the categorizing of testing and development aims and their corresponding outcome measures, a structure was therefore proposed based upon extensive discussions and expert opinion within the group of authors. Another limitation is that the justification of outcome measures was not always mentioned in the articles, such that the correspondence of aims and outcomes had to be inferred by the two reviewers, who independently reviewed the papers in detail and discussed until consensus was reached. Yet, it cannot be excluded that in some cases, these inferences may have deviated from the researchers’ original intentions.

## Conclusion

The current review on prosthesis prototype testing provides an overview of participant characteristics, research protocols, reference values, outcome measures, and how the outcome measures correspond to certain development or testing aims. Considerable heterogeneity was observed in the use of research protocols and outcome measures to validate the stated aims. For future harmonization, ‘benchmarks’ may be developed for evaluation and testing of transfemoral prosthesis prototypes. A core set of reported participant characteristics, testing protocols, and specific and well-founded outcome measures may be established, tailored to the various aims and development phases. The use of such a core set can give greater insights into progress of developments and determine which developments have additional benefits over the state-of-the-art. The overview provided in this review may contribute as initial input towards the development of such a core set.

## Supplementary Information

**Additional file 1. Table S1.** Operationalization of the aim categorization.

**Additional file 2. Table S2.** Methodological aspects of reviewed articles: authors, participant characteristics, research protocol, reference value and aim categories.

**Additional file 3. Table S3.** Reference list indicating which articles contain the corresponding outcome measures for each of the aim categories.

## Data Availability

Not applicable.

## Abbreviations

MPC: Micro-processor controlled
P: Passive
PRISMA: Preferred reporting items for systematic reviews and meta-analyses
PW: Powered
TRL: Technology readiness level
