# Supplementary material for: Testing and evaluation of lower limb prosthesis prototypes in people with a transfemoral amputation: a scoping review on research protocols
Source: J Neuroeng Rehabil. 2023 Jan 12;20:1. doi: 10.1186/s12984-023-01125-8 (PMC9835280; doi:10.1186/s12984-023-01125-8)
Supplement: Supplementary file 2 — Additional file 2. Table S2. Methodological aspects of reviewed articles: authors, participant characteristics, research protocol, reference value and aim categories. [file 12984_2023_1125_MOESM2_ESM.docx]

*Table S2: Methodological aspects of reviewed articles: authors, participant characteristics, research protocol, reference value and aim categories.*

| **Article** |  | **Participants** |  |  |  |  |  | **Protocol** |  |  |  |  |  |  |
| --- | --- | --- | --- | --- | --- | --- | --- | --- | --- | --- | --- | --- | --- | --- |
|  | **N** | **Amputations** | **Reason of amputation** | **Sex and age** | **Level of activity** | **Current knee prosthesis** | **Current foot prosthesis** | **Prototype** | **Familiarization time with the prototype** | **Task** | **Speed** | **Reference value** | **Development aim** | **Testing aim** |
| *Ambrozic et al. 2014(1)* | 3 | 3 Uni Tf | 3 Tr | -, 59.7+-11 years | Self-reported (fair to good), 12, 14, 14 h/day use of prosthesis | - | - | Knee P & Ankle PW: CYBERLEGs system: The robotic prosthesis with knee-ankle energy transfer and intent detection | 5-10 times 6m long walks | Walking (overground with handrails, minimum 20x 6-m) | Self-selected comfortable speed | Intact limb | Energy consumption regarding participant; | - |
| *Andrysek et al. 2011(2)* | 14 | 11 Uni Tf, 1 Uni KD, 2 Uni PFFD | 8 Tr, 3 Tu, 2 Cong, 1 Dys | 12M 2F, 15-67 years (mean: 33.4) | Use prosthesis in community daily, based on self-report | P: Total Knee 2100(2), Total Knee 2000(2), EUK, 3R60, KX06, MauchSNS, 3R106, 3R36, 3R15 without(2) and with spring (2) | P: Axion(3)*^(2)^, Trias*, Seattle Carbon lightfoot*, Multiflex, Vari-Flex(2), Elation, SACH(5)* | P: Simplified automatic stance phase lock knee(SASPL).  With 9 current foot* and 5 Kingsley Seattle Lightfoot | 2 weeks | Walking (overground, 20mWT, 2MWT) | Self-selected comfortable and fast speed | Current prosthesis | Mobility; Prosthesis control; | Mobility; Energy consumption regarding participant; |
| *Andrysek et al. 2009(3)* | 3 | 3 Uni Tf | - | 3M, 15-26 years (17, 26, 15) | - | P: Total Knee 2100&2000 | - | P: Existing single axis knee joint with added motor and sensors | Several 10m passes | Walking (overground, 10 m passes) | Comfortable and fast speed | Between damping conditions, current prosthesis, between speeds | Mobility; Energy handling of prosthesis prototype; Prosthesis control; | Energy handling of prosthesis prototype; Prosthesis control; |
| *Arelekatti et al. 2015(4)* | 2 | 2 Uni Tf | - | - | - | - | - | P: Prototype with, early stance lock, linear spring and damping system | A period | Walking (overground, 2MWT) | - | Current prosthesis | Mobility; Energy consumption regarding participant; Prototype characteristics; | Subjective evaluation; |

| **Article** |  | **Participants** |  |  |  |  |  | **Protocol** |  |  |  |  |  |  |
| --- | --- | --- | --- | --- | --- | --- | --- | --- | --- | --- | --- | --- | --- | --- |
|  | **N** | **Amputations** | **Reason of amputation** | **Sex and age** | **Level of activity** | **Current knee prosthesis** | **Current foot prosthesis** | **Prototype** | **Familiarization time with the prototype** | **Task** | **Speed** | **Reference value** | **Development aim** | **Testing aim** |
| *Arelekatti et al. 2015(5)* | 4 | 4 Uni Tf | - | - | - | P: Polycentric four bar knee | - | P: Prototype with stance lock and damping system | A period | Walking (overground, 2MWT), up and down inclination (25degrees), stairs, outdoors walking on dirt | - | Current prosthesis | Mobility; Energy consumption regarding participant; Prototype characteristics; | Subjective evaluation; |
| *Awad et al. 2016(6)* | 1 | Uni Tf | Inf | M, 53 years | - | P: Passive prosthetic leg | - | PW: Leeds prosthetic knee (LPK) system has active and passive modes based on the energy required, with Echelon prosthetic foot (Chas. A. Blatchford & Sons, UK) | - | Walking (overground with parallel bars) | Self-selected comfortable speed | Current prosthesis, able-bodied (measured) | Energy handling of prosthesis prototype; Effect of powered prosthesis; | Mobility; |
| *Baimyshev et al 2018(7)* | 1 | Uni Tf | - | M, 59 years | - | MPC: Rheo Knee | - | PW: Swing-assist (SA) knee prosthesis with motor to aid flexion in mid swing and extension in late swing, with passive carbon-fiber foot prosthesis | 30 min | Walking (treadmill) | Self-selected comfortable speed (0.9 m/s) +- 20% (3 levels) | Current prosthesis | Prototype characteristics; Effect of powered prosthesis; Prosthesis control; | Mobility; |
| *Bhakta et al. 2018(8)* | 5 | 2 AB, 3 Uni Tf | - | AB: 20, 21 years. TF: 58, 36, 51 years | K3 or higher | - | - | PW: Updated AMPRO3: powered knee and ankle prosthetic device | Until comfortable during walking with the device and had met some common ambulation goals suggested by the prosthetist. | Walking (overground and treadmill) | Overground: self-selected comfortable speed (AB 0.9 m/s, TF ~0.90 m/s); Treadmill: fixed 5 levels (0.63, 0.72, 0.8, 0.89, 0.98, 1.07 m/s) | Able-bodied (measured), Literature values (Winter), between speeds | Subjective evaluation; Mobility; Effect of powered prosthesis; | Mobility; Prosthesis control; |
| *Cao et al. 2018(9)* | 10 | 10 Uni Tf | 10 Tr | 10M, 32-43 years (36, 43, 40, 38, 42, 41, 37, 32, 40, 34) | Walking without support. 10h/day use of prosthesis | - | - | MPC: The prosthetic knee with a hydraulic cylinder, with the Ottobock Triton 1C60 | 3 months | Walking (treadmill) | 3 fixed speeds (0.5, 0.8, 1.1 m/s) | Intact limb, between speeds | Prosthesis control; | Mobility; |

| **Article** |  | **Participants** |  |  |  |  |  | **Protocol** |  |  |  |  |  |  |
| --- | --- | --- | --- | --- | --- | --- | --- | --- | --- | --- | --- | --- | --- | --- |
|  | **N** | **Amputations** | **Reason of amputation** | **Sex and age** | **Level of activity** | **Current knee prosthesis** | **Current foot prosthesis** | **Prototype** | **Familiarization time with the prototype** | **Task** | **Speed** | **Reference value** | **Development aim** | **Testing aim** |
| *Cao et al. 2018(10)* | 12 | 12 Uni Tf | - | 9M 3F, 23-53 years (46, 23, 49, 38, 32, 45, 47, 52, 50, 44, 53, 27) | K3 or higher | P: 3R80 (5), 3R60 (2), CaTech(2), Mauch SNS (3) | - | MPC: i-KNEE with hydraulic dampers for both flexion and extension resistances, with Triton foot | 6 weeks | Walking (treadmill) | Self-selected comfortable walking speed (0.63 m/s); 3 fixed speeds (0.7, 1, 1.4 m/s) | Current prosthesis (with Triton foot), intact limb | Effect of powered prosthesis; | Mobility; |
| *Endo et al. 2019(11)* | 1 | 1 Uni Tf | - | M, 45 years | - | P: 3R80 | - | PW:Powered knee prosthesis with series elastic actuator (PKP-SEA), with Trius foot prosthesis | 10 min (When comfortable) | Walking (treadmill) | Fixed speed (0.8 m/s) | Current prosthesis (with Trius foot) | Mobility; Effect of powered prosthesis; Prototype characteristics; | - |
| *Flynn et al. 2018(12)* | 4 | (with Uni Tf prosthesis) | 3 Tr, 1 Dys | 4M, 63+-11 years | K1 to K3 | - | - | PW: CYBERLEGs prosthesis: highly passive system and capable of providing the high torques and power output for high energy output tasks. | 15 min | Walking (treadmill, 3 min) | Self-selected comfortable speed (for 1 subject 2.2 km/h) | Literature values (Winter and C-leg) | Mobility; | Mobility; |
| *Flynn et al. 2015(13)* | - | Uni Tf | - | - | - | - | - | PW: CYBERLEGs prosthesis: highly passive system and capable of providing the high torques and power output for high energy output tasks. | A short session | Walking (treadmill, 3 min), sit-to-stand and stair climbing | Self-selected comfortable speed | Other: desired position | Mobility; Energy handling of prosthesis prototype; Effect of powered prosthesis; | Mobility; |
| *Flynn et al. 2015(14)* | 3 | 3 Uni Tf | - | - | - | - | - | Knee P & Ankle PW: The CYBERLEGs prosthesis: with knee-ankle energy transfer system | Little training time (20 test runs) | Walking (overground, 10m walkway) | Self-selected comfortable speed (around 1.5 s/stride) | Literature values (Winter), desired position, intact limb | Mobility; Energy consumption regarding participant; Energy handling of prosthesis prototype; | Mobility; Prosthesis control; |
| *Furse et al. 2011(15)* | 1 | Uni Tf | - | -, 18 years | - | - | - | P: The LC Knee: single-axis design with stance-phase control mechanism | One month | Walking (overground, 20m) | Self-selected comfortable (1.13-1.32 m/s) and fast speed (1.53-1.72 m/s) | Between spring conditions, literature values ([hydraulic knee](https://ieeexplore.ieee.org/stamp/stamp.jsp?tp=&arnumber=4376251)) | Mobility; | Mobility; |
| **Article** |  | **Participants** |  |  |  |  |  | **Protocol** |  |  |  |  |  |  |
|  | **N** | **Amputations** | **Reason of amputation** | **Sex and age** | **Level of activity** | **Current knee prosthesis** | **Current foot prosthesis** | **Prototype** | **Familiarization time with the prototype** | **Task** | **Speed** | **Reference value** | **Development aim** | **Testing aim** |
| *Furse et al. 2011(16)* | 7 | 6 Uni Tf, 1 Uni KD | - | 6M 1F, 18-58 years (mean: 36.5) | K3 and K4 | P: Total Knee 2100, Total Knee 2000, EUK, Mauch SNS, KZX06 hydraulic knees, 3R60 | P: Axion, Vari-flex, Elation, Multiflex, Seattle Carbon Lighfoot. | P: Simplified automatic stance phase lock (SASPL) knee with nonfluid-based (NFB) swing-phase control mechanisms, with Seattle Feet (Kingsley) | Two weeks | Walking (overground, 20m) | Self-selected comfortable (1.18 +-0.12 m/s), and fast speed(1.49 +-0.22 m/s) | Current prosthesis (only walking speed), between spring conditions | - | Mobility; Prosthesis control; |
| *Gao et al. 2019(17)* | 1 | Uni Tf | - | M, 30 years | - | MPC: C-Leg compact | - | PW: Robotic electrohydraulic transfemoral prosthesis (RETP) with hydraulic damping adaptation and swing assistance | Until comfortable during walking with the device | Walking (treadmill, 3 min) | Self-selected comfortable speed (0.8 m/s) | Current prosthesis, intact limb | Mobility; Prototype characteristics; | - |
| *Hasenoehrl et al. 2018(18)* | 5 | 5 Uni Tf | 1 Tr, 1 Vas, 1 Can, 2 Sep | 4M 1F, 68.2+-7.7 years (75, 71, 66, 73, 56) | K2 | P: Medi polycentric knee, Medi brake knee, 3R92 brake , 3R93 brake, 3R41 lock knee | - | MPC: Genium with Cenior-Leg ruleset microprocessor controlled knee prosthesis (GCL-MPK) | 4-6 weeks (34, 50,38, 41, 35 days, 39.6+-6.34 days) | Walking (overground, 10mWT, 2MWT) and functional tests (TUG, AMPPro, Berg-balance scale) | Self-selected comfortable and fast speed (comfortable: 0.76 ± 0.08 and 0.73 ± 0.06 m/s for different prosthesis; fast: 0.98 ± 0.144 and 0.88 ± 0.090 m/s for different prosthesis) | Current prosthesis | Mobility; | Subjective evaluation; Mobility; |
| *Hood et al. 2018(19)* | 2 | 1 Uni Tf, 1 AB | - | - | - | - | - | PW: Utah Lightweight Leg: Lightweight powered knee and ankle prosthesis | A few steps overground, and 3x1 min treadmill walking | Walking (treadmill, 4x 4 min) | Fixed speed: AB: 0.76 m/s, Tf: - | Literature values (Winter), between vertical off-sets (Nm/(degrees x kg)) | - | Mobility; Energy consumption regarding participant; |
| *Hoover et al. 2012(20)* | 1 | Uni Tf | Tr | M, 53 years | High level of physical fitness | MPC: C-leg | - | PW: Myoelectric prosthesis prototype: Actively powered transfemoral prosthesis with myoelectric command control | 4x 3hour session until subject demonstrated proficiency in controlling the limb. | Walking (overground, 20m) | Self-selected comfortable speed (2.9 km/h) | No comparison | Prosthesis control; | Prosthesis control; |

| **Article** |  | **Participants** |  |  |  |  |  | **Protocol** |  |  |  |  |  |  |
| --- | --- | --- | --- | --- | --- | --- | --- | --- | --- | --- | --- | --- | --- | --- |
|  | **N** | **Amputations** | **Reason of amputation** | **Sex and age** | **Level of activity** | **Current knee prosthesis** | **Current foot prosthesis** | **Prototype** | **Familiarization time with the prototype** | **Task** | **Speed** | **Reference value** | **Development aim** | **Testing aim** |
| *Jayaraman et al. 2018(21)* | 2 | 1 Uni Tf, 1 Uni KD | 1 Can, 1 Inf s/p tr | M, 25, 58 years | K3, K4 | MPC: Genium 3B1-2=ST, Rheo 3 | P: Multi-Axial Dynamic Response Carbon Fiver Foot (2) | PW: The Vanderbilt Generation 3 powered knee-ankle prosthesis (PKA) with integrated communication between knee and ankle | Up to 12 training sessions | Walking (overground, 7-foot; treadmill, Graded Treadmill Test) | Self-selected comfortable speed | Current prosthesis, intact limb | Mobility; Effect of powered prosthesis; | Mobility; |
| *Khalaf et al. 2018(22)* | 1 | Uni Tf | - | M, 35 years | - | MPC: Freedom Innovations Plie microprocessor-controlled passive knee | P: Triton Vertical Shock foot | Knee PW & Ankle P: Powered regenerative knee joint and a passive ankle joint with semi-active virtual control strategy (SVC). with Ottobock Triton Vertical Shock foot | 2 periods of at least 15 min | Walking (treadmill) | Self-selected comfortable speed **+ - 1.5 m/s (0.6 m/s, 0.75 m/s, and 0.9 m/s)** | Literature values (Winter/Kirtley), sensor data from different positions | Energy handling of prosthesis prototype; Prosthesis control; Effect of powered prosthesis; | - |
| *Lambrecht et al. 2009(23)* | 3 | 3 UniTf | - | - | - | - | - | PW: The semi-active knee with active (hydraulic) and passive mode | Until comfortable with the weight and feel of the device. | Walking (treadmill; overground between parallel bars), Ramps and stairs (with handrails) | Various speeds | No comparison | Mobility; Effect of powered prosthesis; | - |
| *Lawson et al. 2014(24)* | 3 | 3 UniTf | 2 Tr, 1 Can | , 25, 24, 46 years | - | - | - | PW: Mechanically separable powered knee and ankle joints to ensure height adjustment, and alignment norms | - | Walking (overground) | Self-selected speed | Intact limb | Mobility; | Mobility; Prosthesis control; |
| *Lee et al. 2020(25)* | 1 | Uni Tf | - | M, | - | MPC: Rheo knee | - | PW: Stance-controlled swing-assisted (SCSA) prosthesis prototype with low-impedance power. | 30-60 minutes | Walking (treadmill), perturbed walking (treadmill) | Self-selected comfortable speed (0.8 m/s) | Current prosthesis | Mobility; Effect of powered prosthesis; | Mobility; |

| **Article** |  | **Participants** |  |  |  |  |  | **Protocol** |  |  |  |  |  |  |
| --- | --- | --- | --- | --- | --- | --- | --- | --- | --- | --- | --- | --- | --- | --- |
|  | **N** | **Amputations** | **Reason of amputation** | **Sex and age** | **Level of activity** | **Current knee prosthesis** | **Current foot prosthesis** | **Prototype** | **Familiarization time with the prototype** | **Task** | **Speed** | **Reference value** | **Development aim** | **Testing aim** |
| *Lee et al. 2009(26)* | 5 | 5 Uni Tf | 5 Tr | 5M, 47.6+-2.2 years (54, 41, 45, 50, 48) | - | P: Four-bar(4), Single(1) knee prosthesis | - | Knee MCP: Five-bar-linkage mechanism with a pneumatic mechanism, with two different foot Foot P: Ankle X with fixed angle in neutral; Ankle Z with 15 degrees of dorsiflexion and 25 degrees of plantarflexion | 2 days | Walking (overground) | Fixed speed (+- 0.7 m/s) | Two foot prosthesis and literature values (own research amputee walking) | - | Mobility; |
| *Lenzi et al. 2018(27)* | 2 | 2Uni Tf | - | 2M, 28, 68 years | K3 | - | - | PW: Hybrid knee with spring-damper system and an electric motor and transmission system | 45 min ambulation on stairs, and about 30 strides | Walking (overground) and stairs ascent/descent, and transitions between walking in the passive mode and climbing stairs in the active mode. | Self-selected speed | Literature values (Able bodied (Riener et al. 2002), and commercial available passive knee (Segal et al. 2006&Lawson et al. (2012)) | Mobility; Prototype characteristics; Effect of powered prosthesis; | Mobility; |
| *Li et al. 2019(28)* | 1 | Uni Tf | Can | M, 25 years | Experienced at prosthesis ambulation. | P: Freedom passive prosthetic leg (Tehlin) | - | PW: Powered knee and ankle prosthesis | 5 test sessions | Walking (overground), ramp and stairs ascent/descent | Self-selected, slow and fast walking | Desired referenced trajectories | Mobility; Prosthesis control; | Mobility; |
| *Li et al. 2019(29)* | 10 | 10 Uni Tf | 7 Tr, 3 Disease | 7M 3F, 20-45 years (20, 26, 23, 35, 38, 45, 33, 44, 21, 32) | K3 or higher | - | - | MPC: i-KNEE: Hydraulic damper with two motorized valves to generate knee joint resistance and a steel spring to store energy, with a triton foot | At least 3 days | Walking (treadmill) | 5 fixed speeds (0.5, 0.7, 0.9, 1.1, 1.3 m/s) | Between speeds, different knee prosthesis (the C-Leg, the Rheo Knee and the Mauch all with Triton foot (Ottobock)) | - | Mobility; Energy consumption regarding participant; |
| *Martinez-Villalpando et al. 2011(30)* | 2 | 1 AB, 1 Uni Tf | - | 2M, | K3 | MPC: C-leg | - | PW: Biomimetic active knee prosthesis with two unidirectional series elastic actuators (SEAs), with prosthetic foot Flex-Foot LP-VariFlex | 15 min | TF: Walking (overground, 10m walkway and indoor track), Able-bodied: walking (Treadmill) | TF: Self-selected comfortable speed (1.3 m/s), AB: 3 fixed speeds (0.9, 1.1, 1.3 m/s) | Current prosthesis | Energy handling of prosthesis prototype; Prosthesis control; Effect of powered prosthesis; | AB: Prosthesis control; TF: Energy consumption regarding participant; |

| **Article** |  | **Participants** |  |  |  |  |  | **Protocol** |  |  |  |  |  |  |
| --- | --- | --- | --- | --- | --- | --- | --- | --- | --- | --- | --- | --- | --- | --- |
|  | **N** | **Amputations** | **Reason of amputation** | **Sex and age** | **Level of activity** | **Current knee prosthesis** | **Current foot prosthesis** | **Prototype** | **Familiarization time with the prototype** | **Task** | **Speed** | **Reference value** | **Development aim** | **Testing aim** |
| *Murthy Arelekatti et al. 2018(31)* | 4 | 4 Uni Tf | - | 4M, >35 years | - | P: Jaipur-Stanford four-bar polycentric knee joint (3), single axis knee (1) | - | P: Fully passive prosthetic knee mechanism with early stance lock and differential friction damping system | A period | Walking (overground, 2MWT), up/down slope (25 deg), stairs, uneven terrain | - | Current prosthesis | Mobility; Prototype characteristics; | Subjective evaluation; |
| *Ochoa-Diaz et al. 2014(32)* | 1 | Uni | - | M, 35 years | - | P: 3R80 | - | P: Variable-damping prosthesis: low cost four-bar polycentric system with variable-damping control. | Had worn the knee prosthesis before the testing | Walking (treadmill) | Comfortable speed | No comparison | Mobility; Prototype characteristics; | Mobility; |
| *Pandit et al. 2018(33)* | 1 | Uni Tf | Tr | M, 24 years | Above K3 level | MPC: single axis extension assisting prosthetic knee joint | P: SACH-foot | MPC: Passive prosthesis with magneto-rheological (MR) damping system and electronic control, with a SACH foot | After tuning of both control levels, a number of training sessions. Until subject actively started to walk on level ground | Walking (overground, 80-feet walkway) | Self-selected comfortable speed | No comparison | Mobility; Prototype characteristics; | - |
| *Pfeifer et al. 2015(34)* | 1 | Uni Tf | - | , 44 years | - | - | - | P: ANGELLA, Angle-dependent Elastic actuator, variant of Series (Visco-) Elastic Actuator (SVA), with Vari-flex foot | 5 min | Walking (treadmill) | Self-selected speed (2.2 km/h) | Reference knee moment (unspecified) | Mobility; Prosthesis control; Prototype characteristics; | Mobility; |
| *Ramakrishnan et al. 2017(35)* | 1 | Uni Tf | - | F, 37 years | High functioning | P: Total knee 2000 | - | P: The anatomically 3D printed scalable transfemoral prosthetic knee with gear-mesh coupling and weight-actuated locking mechanism | - | Walking (treadmill) | Self-selected speed (1.4 m/s) | Ossur Total Knee 2000, intact limb | Energy consumption regarding participant; Prototype characteristics; | Mobility; |
| *Rouse et al. 2014(36)* | 1 | Uni Tf | - | M, 48 years | - | MPC: MPC knee | P: Vertical shock energy return prosthetic foot | PW: CSEA knee: Powered knee prosthesis with clutchable series-elastic actuator (CSEA) including low-power clutch in parallel with an electric motor, with powered ankle prosthesis (BioM) | - | Walking (treadmill) | Self-selected speed (1.3 m/s) | Reference torque-angle profile (weight-matched standardized biological profile) | Mobility; Energy handling of prosthesis prototype; | Mobility; Energy handling of prosthesis prototype; |

| **Article** |  | **Participants** |  |  |  |  |  | **Protocol** |  |  |  |  |  |  |
| --- | --- | --- | --- | --- | --- | --- | --- | --- | --- | --- | --- | --- | --- | --- |
|  | **N** | **Amputations** | **Reason of amputation** | **Sex and age** | **Level of activity** | **Current knee prosthesis** | **Current foot prosthesis** | **Prototype** | **Familiarization time with the prototype** | **Task** | **Speed** | **Reference value** | **Development aim** | **Testing aim** |
| *Sharma et al. 2020(37)* | 1 | Uni Tf | - | M, 25 years | - | P: passive spring-based linkage knee mechanism prosthetic leg | - | MPC: Simplefied, robust transfemural leg, with magnetorheological-damper (from Lord Corporation RD-8041), simplified control strategy and cost-effective control approach. | 10 daylong sessions | Walking (overground, 10m) | Self-selected speed | Intact limb | Prosthesis control; | Mobility; |
| *Sun et al. 2018(38)* | 1 | Uni Tf | - | M, 30 years | - | - | - | PW: Robotic knee-ankle prosthesis with elastic actuators, variable transmission ration of each joint and ability to adjust shank length and alignment between knee and ankle | - | Walking | - | No comparison | Prototype characteristics; Effect of powered prosthesis; | - |
| *Sun et al. 2018(39)* | 1 | Uni Tf | - | M, 30 years | - | - | - | PW: SuKnee: Novel robotic prosthetic knee with variable transmission mechanism | - | Walking (treadmill) and sit-to-stand | 4 km/h | Literature values (Winter), control trajectory | Mobility; Energy handling of prosthesis prototype; Prototype characteristics; Effect of powered prosthesis; | - |
| *Sup et al. 2009(40)* | 1 | Uni Tf | - | M, 20 years | - | MPC: C-leg | P: Freedom Renegade | PW: Electrically powered self-contained active knee and ankle prosthesis with torque-based control framework | - | Walking (overground, 50m; treadmill for parameter tuning) | Self-selected comfortable speed (overground: 4.1 km/h --> 5.1 km/h; treadmill: 2.8 km/h +- 15%) | Literature values (Winter) | Mobility; Effect of powered prosthesis; | Mobility; Energy handling of prosthesis prototype; |
| *Torrealba et al. 2010(41)* | 3 | 1 Uni Tf, 2 AB (1 with AB-adaptor) | - | - | - | - | - | MPC: Intelligent knee prosthesis with gait cycle characterization | - | Walking (treadmill) | Self-selected speed | Reference knee angle (unspecified) | Prosthesis control; | - |

| **Article** |  | **Participants** |  |  |  |  |  | **Protocol** |  |  |  |  |  |  |
| --- | --- | --- | --- | --- | --- | --- | --- | --- | --- | --- | --- | --- | --- | --- |
|  | **N** | **Amputations** | **Reason of amputation** | **Sex and age** | **Level of activity** | **Current knee prosthesis** | **Current foot prosthesis** | **Prototype** | **Familiarization time with the prototype** | **Task** | **Speed** | **Reference value** | **Development aim** | **Testing aim** |
| *Torrealba et al. 2009(42)* | 1 | Uni Tf | - | - | - | - | - | MPC: The knee prosthesis with magneto-rheological actuator and gait cycle characterization | - | Walking (treadmill) | 2 km/h | No comparison | Prototype characteristics; | - |
| *Unal et al. 2013(43)* | 1 | Uni Tf | - | - | - | - | - | PW: WalkMECH: Fully-passive transfemoral prosthesis with three elastic elements and energetic coupling | - | Walking (treadmill) | Self-selected speed (4.6 km/h) | Simulation, literature values (Winter) | Mobility; Energy consumption regarding participant; Prototype characteristics; | - |
| *Valencia et al. 2018(44)* | 1 | Uni Tf | - | M, 48 years | - | P: Monocentric Otto Bock prosthesis | - | P: Monocentric knee prosthesis | - | Walking and running | - | No comparison | Subjective evaluation; Prototype characteristics; | - |
| *Williams et al. 2016(45)* | 5 | 5 Uni Tf | 3 Tr, 1 Vas, 1 Can | , inclusion criteria: 20-70 years | Inclusion criteria: K3 or above | MPC: C-leg | P: Low Profile Vari-Flex(2), College Park Trustep, Trition, Flex Walk | PW: VI Knee: Variable impedance knee prosthesis (VI Knee) with two series elastic actuators to control knee impedance, with own foot | 30-60 minutes | Walking (overground, 10m walkway) | Fixed speeds (1, 1.25 m/s) | Between speeds, current prosthesis | Mobility; Prosthesis control; Effect of powered prosthesis; | Mobility; |
| *Williams et al. 2016(46)* | 5 | 5 Uni Tf | 3 Tr, 1 Vas, 1 Can | , 37-61 years (37, 49, 55, 52, 61) | Inclusion criteria: K3 or above | MPC: C-leg | P: Low Profile Vari-Flex, Low Profile Re-Flex VSP, Collega Park Trustep, Trition, Flex Walk | PW: VI Knee: Variable impedance knee prosthesis (VI Knee) with two series elastic actuators to control knee impedance, with own foot | 30 min | Walking (overground, hallway and 10 walkway) | Fixed speeds (1, 1.25 m/s) | Between speeds, current prosthesis | Mobility; Energy consumption regarding participant; Prosthesis control; Effect of powered prosthesis; | Mobility; Energy consumption regarding participant; |
| *Xavier et al. 2017(47)* | - | - | - | - | - | - | - | MPC: Low- cost and low-weight prosthesis for transfemoral amputation leg using myoelectric signals | - | Walking | - | Sound limb (unclear whether able-bodied or amputees) | Mobility; Prototype characteristics; | - |

| **Article** |  | **Participants** |  |  |  |  |  | **Protocol** |  |  |  |  |  |  |
| --- | --- | --- | --- | --- | --- | --- | --- | --- | --- | --- | --- | --- | --- | --- |
|  | **N** | **Amputations** | **Reason of amputation** | **Sex and age** | **Level of activity** | **Current knee prosthesis** | **Current foot prosthesis** | **Prototype** | **Familiarization time with the prototype** | **Task** | **Speed** | **Reference value** | **Development aim** | **Testing aim** |
| *Yokogushi et al. 2004(48)* | 13 | 3 Uni Tf, 10 AB | 3 Tr | TF: 3M, 28.0 +- 1.5 years AB: 3M, 22.0 +- 1.5 years | - | P: 3R60 | - | MPC: Polycentric knee with 4-bar linkage, intermediate links, a hydraulic unit working during the stance phase, and a pneumatic unit controlled by a microprocessor working during the swing phase | 30 min | Walking (overground, on a platform) | Fixed cadence (88, 96, 104 steps/min) | Current prosthesis, able-bodied (measured), between speeds | Mobility; | Subjective evaluation; Mobility; |

*Uni: Unilateral, Tf: Transfemoral, KD: Knee disarticulate, PFFD: Proximal femoral focal deficiency, AB: Able-bodied, Tr: Trauma, Tu: Tumor, Cong: Congenital, Dys: Dysvascular, Inf: Infection, Vas: Vascular, Can: Cancer, Sep: Sepsis, M: male, F: Female, P: passive, MPC: micro-processor controlled, PW: powered, -: Not specified*

**References:**

1. Ambrozic L, Gorsic M, Geeroms J, Flynn L, Molino Lova R, Kamnik R, et al. CYBERLEGs: A user-oriented robotic transfemoral prosthesis with whole-body awareness control. Ieee Robot Autom Mag. 2014;21(4):82-93.

2. Andrysek J, Klejman S, Torres-Moreno R, Heim W, Steinnagel B, Glasford S. Mobility function of a prosthetic knee joint with an automatic stance phase lock. Prosthet Orthot Int. 2011;35(2):163-70.

3. Andrysek J, Liang T, Steinnagel B. Evaluation of a prosthetic swing-phase controller with electrical power generation. IEEE Trans Neural Syst Rehabil Eng. 2009;17(4):390-6.

4. Arelekatti VNM, Winter AG, editors. Design of a fully passive prosthetic knee mechanism for transfemoral amputees in India. IEEE International Conference on Rehabilitation Robotics; 2015.

5. Arelekatti VNM, Winter AG, editors. Design of mechanism and preliminary field validation of low-cost, passive prosthetic knee for users with transfemoral amputation in India. Proceedings of the ASME Design Engineering Technical Conference; 2015.

6. Awad MI, Abouhossein A, Dehghani-Sanij AA, Richardson R, Moser D, Zahedi S, et al. Towards a Smart Semi-Active Prosthetic Leg: Preliminary Assessment and Testing. Ifac Papersonline. 2016;49(21):170-6.

7. Baimyshev A, Lawson B, Goldfarb M. Design and Preliminary Assessment of Lightweight Swing-Assist Knee Prosthesis. Annu Int Conf IEEE Eng Med Biol Soc. 2018;2018:3198-201.

8. Bhakta K, Camargo J, Young AJ, editors. Control and experimental validation of a powered knee and ankle prosthetic device. ASME 2018 Dynamic Systems and Control Conference, DSCC 2018; 2018.

9. Cao W, Yu H, Meng Q, Chen W, Li S. Plantar pressure analysis of above-knee amputee with a developed microprocessor-controlled prosthetic knee. Acta Bioeng Biomech. 2018;20(4):33-40.

10. Cao W, Yu H, Zhao W, Meng Q, Chen W. The comparison of transfemoral amputees using mechanical and microprocessor- controlled prosthetic knee under different walking speeds: A randomized cross-over trial. Technol Health Care. 2018;26(4):581-92.

11. Endo K, Takeshima H, Tawara T, editors. Development of Powered Knee Prosthesis with Small-Scale, Light-Weight, and Affordable Series-Elastic Actuator, and its Preliminary Walking Test. Proceedings of the IEEE 2019 9th International Conference on Cybernetics and Intelligent Systems and Robotics, Automation and Mechatronics, CIS and RAM 2019; 2019.

12. Flynn L, Geeroms J, Jimenez-Fabian R, Heins S, Vanderborght B, Munih M, et al. The Challenges and Achievements of Experimental Implementation of an Active Transfemoral Prosthesis Based on Biological Quasi-Stiffness: The CYBERLEGs Beta-Prosthesis. Front Neurorobot. 2018;12:80.

13. Flynn L, Geeroms J, Jimenez-Fabian R, Vanderborght B, Lefeber D, editors. CYBERLEGS Beta-Prosthesis active knee system. IEEE International Conference on Rehabilitation Robotics; 2015.

14. Flynn L, Geeroms J, Jimenez-Fabian R, Vanderborght B, Vitiello N, Lefeber D. Ankle-knee prosthesis with active ankle and energy transfer: Development of the CYBERLEGs Alpha-Prosthesis. Robot Auton Syst. 2015;73:4-15.

15. Furse A, Cleghorn W, Andrysek J. Development of a low-technology prosthetic swing-phase mechanism. J Med Biol Eng. 2011;31(2):145-50.

16. Furse A, Cleghorn W, Andrysek J. Improving the gait performance of non-fluid-based swing-phase control mechanisms in transfemoral prostheses. IEEE Trans Biomed Eng. 2011;58(8).

17. Gao S, Wang C, Zhu J, Mai J, Wang Q, editors. Hydraulic Damping and Swing Assistance Control of A Robotic Electrohydraulic Transfemoral Prosthesis: Preliminary Results. Proceedings of IEEE Workshop on Advanced Robotics and its Social Impacts, ARSO; 2019.

18. Hasenoehrl T, Schmalz T, Windhager R, Domayer S, Dana S, Ambrozy C, et al. Safety and function of a prototype microprocessor-controlled knee prosthesis for low active transfemoral amputees switching from a mechanic knee prosthesis: a pilot study. Disabil Rehabil Assist Technol. 2018;13(2):157-65.

19. Hood SA, Lenzi T. Preliminary Analysis Of Positive Knee Energy Injection In A Transfemoral Amputee Walking With A Powered Prosthesis. Annu Int Conf IEEE Eng Med Biol Soc. 2018;2018:1821-4.

20. Hoover CD, Fulk GD, Fite KB. The Design and Initial Experimental Validation of an Active Myoelectric Transfemoral Prosthesis. J Med Devices. 2012;6(1).

21. Jayaraman C, Hoppe-Ludwig S, Deems-Dluhy S, McGuire M, Mummidisetty C, Siegal R, et al. Impact of Powered Knee-Ankle Prosthesis on Low Back Muscle Mechanics in Transfemoral Amputees: A Case Series. Front Neurosci. 2018;12:134.

22. Khalaf P, Warner H, Hardin E, Richter H, Simon D, editors. Development and experimental validation of an energy regenerative prosthetic knee controller and prototype. ASME 2018 Dynamic Systems and Control Conference, DSCC 2018; 2018.

23. Lambrecht BGA, Kazerooni H, editors. Design of a semi-active knee prosthesis. Proceedings - IEEE International Conference on Robotics and Automation; 2009.

24. Lawson BE, Mitchell J, Truex D, Shultz A, Ledoux E, Goldfarb M. A robotic leg prosthesis: Design, control, and implementation. Ieee Robot Autom Mag. 2014;21(4):70-81.

25. Lee JT, Bartlett HL, Goldfarb M. Design of a Semipowered Stance-Control Swing-Assist Transfemoral Prosthesis. Ieee-Asme T Mech. 2020;25(1):175-84.

26. Lee S, Hong J. The effect of prosthetic ankle mobility in the sagittal plane on the gait of transfemoral amputees wearing a stance phase controlled knee prosthesis. Proc Inst Mech Eng H. 2009;223(2):263-71.

27. Lenzi T, Cempini M, Hargrove L, Kuiken T. Design, development, and testing of a lightweight hybrid robotic knee prosthesis. Int J Robot Res. 2018;37(8):953-76.

28. Li Q, Chen S, Xu C, Chu X, Li Z, editors. Design, Control and Implementation of a Powered Prosthetic Leg. 2018 11th International Workshop on Human Friendly Robotics, HFR 2018; 2019.

29. Li S, Cao W, Yu H, Meng Q, Chen W. Physiological parameters analysis of transfemoral amputees with different prosthetic knees. Acta Bioeng Biomech. 2019;21(3):135-42.

30. Martinez-Villalpando EC, Mooney L, Elliott G, Herr H. Antagonistic active knee prosthesis. A metabolic cost of walking comparison with a variable-damping prosthetic knee. Annu Int Conf IEEE Eng Med Biol Soc. 2011;2011:8519-22.

31. Murthy Arelekatti VN, Winter VAG. Design and preliminary field validation of a fully passive prosthetic knee mechanism for users with transfemoral amputation in India. Journal of Mechanisms and Robotics. 2018;10(3).

32. Ochoa-Diaz C, Rocha TS, De Levy Oliveira L, Paredes MG, Lima R, Padilha A, et al., editors. An above-knee prosthesis with magnetorheological variable-damping. Proceedings of the IEEE RAS and EMBS International Conference on Biomedical Robotics and Biomechatronics; 2014.

33. Pandit S, Godiyal AK, Vimal AK, Singh U, Joshi D, Kalyanasundaram D. An Affordable Insole-Sensor-Based Trans-Femoral Prosthesis for Normal Gait. Sensors (Basel). 2018;18(3).

34. Pfeifer S, Pagel A, Riener R, Vallery H. Actuator with angle-dependent elasticity for biomimetic transfemoral prostheses. Ieee-Asme T Mech. 2015;20(3):1384-94.

35. Ramakrishnan T, Schlafly M, Reed KB. Evaluation of 3D printed anatomically scalable transfemoral prosthetic knee. IEEE Int Conf Rehabil Robot. 2017;2017:1160-4.

36. Rouse EJ, Mooney LM, Herr HM. Clutchable series-elastic actuator: Implications for prosthetic knee design. Int J Robot Res. 2014;33(13):1611-25.

37. Sharma R, Singh D, Tiwari A, Joshi D. User-feedback based robust and simplified damping control for affordable transfemoral prosthesis. Electron Lett. 2020;56(8):366-7.

38. Sun X, Sugai F, Okada K, Inaba M, editors. Design and Control of a Novel Robotic Knee-Ankle Prosthesis System. Proceedings of the IEEE RAS and EMBS International Conference on Biomedical Robotics and Biomechatronics; 2018.

39. Sun X, Sugai F, Okada K, Inaba M, editors. Variable Transmission Series Elastic Actuator for Robotic Prosthesis. Proceedings - IEEE International Conference on Robotics and Automation; 2018.

40. Sup F, Varol HA, Mitchell J, Withrow TJ, Goldfarb M. Preliminary Evaluations of a Self-Contained Anthropomorphic Transfemoral Prosthesis. IEEE ASME Trans Mechatron. 2009;14(6):667-76.

41. Torrealba RR, Pérez-D'Arpino C, Cappelletto J, Fermín-León L, Fernández-López G, Grieco JC, editors. Through the development of a biomechatronic knee prosthesis for transfemoral amputees: Mechanical design and manufacture, human gait characterization, intelligent control strategies and tests. Proceedings - IEEE International Conference on Robotics and Automation; 2010.

42. Torrealba RR, Zambrano LA, Andara E, Fernández-López G, Grieco JC, editors. Medium-cost electronic prosthetic knee for transfemoral amputees: A medical solution for developing countries. IFMBE Proceedings; 2009.

43. Unal R, Klijnstra F, Burkink B, Behrens SM, Hekman EE, Stramigioli S, et al. Modeling of WalkMECH: a fully-passive energy-efficient transfemoral prosthesis prototype. IEEE Int Conf Rehabil Robot. 2013;2013:6650406.

44. Valencia F, Ortiz D, Ojeda D, editors. Design and testing of low-cost knee prosthesis. 2017 IEEE 2nd Ecuador Technical Chapters Meeting, ETCM 2017; 2018.

45. Williams MR, D'Andrea S, Herr HM. Impact on gait biomechanics of using an active variable impedance prosthetic knee. J Neuroeng Rehabil. 2016;13(1):54.

46. Williams MR, Herr H, D'Andrea S. Metabolic effects of using a variable impedance prosthetic knee. J Rehabil Res Dev. 2016;53(6):1079-88.

47. Xavier B, Mayra C, Johanna T, De La Cruz D, Loza D, Corella J, editors. Low cost mechatronics prototype prosthesis for transfemoral amputation controled by myolectric signals. Lecture Notes in Engineering and Computer Science; 2017.

48. Yokogushi K, Narita H, Uchiyama E, Chiba S, Nosaka T, Yamakoshi K. Biomechanical and clinical evaluation of a newly designed polycentric knee of transfemoral prosthesis. J Rehabil Res Dev. 2004;41(5):675-82.
